# Genetic, Physical and Comparative Mapping of the Powdery Mildew Resistance Gene *Pm21* Originating from *Dasypyrum villosum*

**DOI:** 10.3389/fpls.2017.01914

**Published:** 2017-11-07

**Authors:** Huagang He, Yaoyong Ji, Shanying Zhu, Bin Li, Renhui Zhao, Zhengning Jiang, Tongde Bie

**Affiliations:** ^1^School of Food and Biological Engineering, Jiangsu University, Zhenjiang, China; ^2^School of Environment, Jiangsu University, Zhenjiang, China; ^3^Key Laboratory of Wheat Biology and Genetic Improvement on Low and Middle Yangtze River Valley Wheat Region (Ministry of Agriculture), Yangzhou Academy of Agricultural Sciences, Yangzhou, China

**Keywords:** *Dasypyrum villosum*, *Pm21*, powdery mildew resistance, genetic mapping, physical mapping, comparative mapping

## Abstract

*Pm21*, originating from wheat wild relative *Dasypyrum villosum*, confers immunity to all known races of *Blumeria graminis* f. sp. *tritici* (*Bgt*) and has been widely utilized in wheat breeding. However, little is known on the genetic basis of the *Pm21* locus. In the present study, four seedling-susceptible *D. villosum* lines (DvSus-1 ∼ DvSus-4) were identified from different natural populations. Based on the collinearity among genomes of *Brachypodium distachyon, Oryza*, and *Triticeae*, a set of 25 gene-derived markers were developed declaring the polymorphisms between DvRes-1 carrying *Pm21* and DvSus-1. Fine genetic mapping of *Pm21* was conducted by using an extremely large F_2_ segregation population derived from the cross DvSus-1/DvRes-1. Then *Pm21* was narrowed to a 0.01-cM genetic interval defined by the markers 6VS-08.4b and 6VS-10b. Three DNA markers, including a resistance gene analog marker, were confirmed to co-segregate with *Pm21*. Moreover, based on the susceptible deletion line Y18-S6 induced by ethyl methanesulfonate treatment conducted on Yangmai 18, *Pm21* was physically mapped into a similar interval. Comparative analysis revealed that the orthologous regions of the interval carrying *Pm21* were narrowed to a 112.5 kb genomic region harboring 18 genes in *Brachypodium*, and a 23.2 kb region harboring two genes in rice, respectively. This study provides a high-density integrated map of the *Pm21* locus, which will contribute to map-based cloning of *Pm21*.

## Introduction

Common wheat (*Triticum aestivum* L.) is the most widely grown cereal crop occupying ∼17% of all cultivated land of the world and provides ∼20% of the calories consumed by humankind (Fu et al., 2009). However, wheat production is seriously threatened by various diseases, such as head scab, rusts, and powdery mildew. Wheat powdery mildew caused by the obligate biotrophic fungal pathogen *Blumeria graminis* f. sp. *tritici* (*Bgt*) is one of the most important factors leading to yield losses. Development of resistant varieties using powdery mildew resistance (*Pm*) genes is an effective, economical and environmental-friendly way to reduce yield losses caused by *Bgt*. Up to now, 58 resistant genes have been formally designated (*Pm1* ∼*Pm58*). Among them, some *Pm* genes were identified from the species in the tertiary gene pool, including *Pm7, Pm8, Pm17*, and *Pm20* from *Secale cereal, Pm40* and *Pm43* from *Thinopyrum intermedium*, and *Pm21* and *Pm55* from *Dasypyrum villosum* (McIntosh et al., 2013; Wiersma et al., 2017).

*Dasypyrum villosum* Candargy (2*n* = 14, VV), a diploid wild relative of common wheat, has been an important resource for wheat improvement which provides resistance to multiple wheat diseases, such as powdery mildew, rusts, and eyespot (Chen et al., 2013). The powdery mildew resistance gene *Pm21*, located on the short arm of chromosome 6V (6VS) of *D. villosum*, confers immunity to all known *Bgt* races (Chen et al., 1995; Cao et al., 2011). After emergence of highly virulent *Bgt* isolates, such as *Bgt* YZ01 (He et al., 2016), wheat varieties carrying *Pm2a* or *Pm4a* broadly planted are gradually losing their resistance in the middle and lower reaches of the Yangtze River Valley, the most rampant area of powdery mildew in China. As an important replacement, varieties carrying the translocated chromosome T6AL.6VS are being planted more widely in this region (Bie et al., 2015). In the other major wheat regions in China, the number of the varieties integrating *Pm21* is also growing fast. To better utilize *Pm21*, clarification of the genetic basis and functional mechanism of *Pm21* becomes very important and urgent.

Previously, *Pm21* has been physically mapped to the bin 6VS FL0.45–0.58 by using the resistant deletion line del.6VS-1 (FL0.58) and the susceptible deletion line del.6VS-2 (FL0.45) (Cao et al., 2011). Recently, several candidate genes located in the bin FL0.45–0.58 were found and verified to be required by *Pm21*-mediated resistance to powdery mildew (Cao et al., 2011; He et al., 2016). However, the exact genetic relationships of these genes and *Pm21* remain unclear. To date, there are still two major obstacles for genetic mapping of *Pm21*. First, *D. villosum* chromosome 6VS cannot recombine with homoeologous wheat chromosomes. Second, there is neither natural nor artificial induced susceptible mutant of *D. villosum* that could be used for genetic mapping.

To fulfill genetic mapping of *Pm21* at the diploid level, an attempt was made to obtain susceptible *D. villosum* line(s). Fortunately, by screening more than 100 *D. villosum* accessions collected from different germplasm resources organizations, we found four *D. villosum* accessions susceptible to wheat powdery mildew. Using an F_2_ population derived from the cross between a resistant *D. villosum* line and a susceptible one, fine genetic mapping of *Pm21* was initiated. In combination with physical and comparative mapping, a high-density integrated map of *Pm21* was successfully constructed. This study will contribute to map-based cloning of *Pm21* and understanding of its functional mechanism of broad-spectrum resistance.

## Materials and Methods

### Plant Materials

A collection of 110 accessions of *D. villosum* were kindly provided by Germplasm Resources Information Network (GRIN) (51), GRIN Czech (16), Genebank Information System of the IPK Gatersleben (GBIS-IPK) (35), Nordic Genetic Resource Center (NordGen) (7), and the Cytogenetics Institute, Nanjing Agricultural University (CI-NAU) (1). The susceptible *D. villosum* line DvSus-1 was crossed with the resistant line DvRes-1 carrying *Pm21* (**Table 1**), and the generated F_2_ population containing 10,536 individuals was used for genetic analysis. The powdery mildew resistant wheat variety Yangmai 18, carrying a pair of translocated chromosomes T6AL.6VS, and the susceptible variety Yangmai 9 were both developed in Yangzhou Academy of Agricultural Sciences (YAAS). About 2,000 seeds of Yangmai 18 were treated with 0.8% ethyl methanesulfonate (EMS), and 1,216 M_2_ families were generated to use for screening of mutants susceptible to powdery mildew.

**Table 1:** Origin of *Dasypyrum villosum* lines used in this study.

Line	Powdery mildew response	Origin	Original accession	Provider
DvRes-1	Resistant	United Kingdom	Unknown	CI-NAU
DvSus-1	Susceptible	Greece	GRA 2738	GBIS-IPK
DvSus-2	Susceptible	Unknown	GRA 962	GBIS-IPK
DvSus-3	Susceptible	Italy	GRA 1105	GBIS-IPK
DvSus-4	Susceptible	Former Soviet Union	PI 598390	GRIN

*CI-NAU, the Cytogenetics Institute, Nanjing Agricultural University; GBIS-IPK, Genebank Information System of the IPK Gatersleben; GRIN, Germplasm Resources Information Network.*

### Evaluation of Powdery Mildew Resistance

The *D. villosum* accessions, F_1_ and F_2_ individuals of the cross DvSus-1/DvRes-1, and M_2_ individuals of EMS-induced Yangmai 18 at one-leaf stage were inoculated with *Bgt* isolate YZ01, a predominant race collected from Yangzhou (He et al., 2016), by dusting from sporulating susceptible variety Yangmai 9 and powdery mildew responses were assessed at 7 days post-inoculation in greenhouse. Due to that *Pm21* confers immunity to *Bgt* isolate YZ01, powdery mildew responses can be simply divided into two types, resistant and susceptible.

### Development of DNA Markers

Polymorphic DNA markers between the resistant line DvRes-1 and the susceptible line DvSus-1 were developed using the CISP-IS (conserved intron scanning primer combined with intron sequencing) strategy based on the collinearity relationship between *Brachypodium*, rice and *Triticeae* species (He et al., 2013). The common genes between *Brachypodium* and rice were used to search their homologous full-length cDNAs or ESTs of wheat, barley, *Aegilops* or other Triticeae crops deposited in GenBank database. After alignment of these sequences by the Clustalw tool, primers were designed according to the conserved extron sequences and used to amplify the corresponding intronic regions of the parents DvRes-1 and DvSus-1. After T/A cloning and sequencing of the target fragments, the flanking conserved sequences of the variant introns were further used to design primers and screen polymorphic markers. DNA markers used for physical mapping were also developed using the same strategy. The details of polymorphic markers used in this study were listed in Supplementary Tables S1, S2.

### Genomic DNA Isolation and Marker Analysis

Genomic DNA with high-quality was isolated from fresh leaves of the seedlings by the CTAB method (Murray and Thompson, 1980). For high-throughput marker analysis, a simple and rapid boiling method for crude DNA extraction was newly developed in this study. A leaf segment (about 1 cm long) was grinded by glass rod in a 1.5-ml tube. Then, added 150 μl of DNA protection solution (50 mM Tris–HCl, 25 mM EDTA, pH 8.0) into the tube and boiled at 90°C for 10 min. After centrifugation at 10,000 rpm for 1 min, the supernatant was directly used as DNA template for PCR amplification. PCR amplification was performed in a Peltier thermal cycler (Bio-Rad, United States) in 25 μl volume containing 1 × PCR buffer (Mg^2+^ free), 2.7 mM of MgCl_2_, 0.2 mM of each dNTP, 2 μM of each primer, 1 unit of *Taq* DNA polymerase, and 1 μl of DNA template. PCR was carried out with an initial denaturation at 94°C for 3 min, 35 cycles of 20 s at 94°C, 30 s at 60°C, 1 min at 72°C, and a final extension for 5 min at 72°C. PCR products were separated in 6% ∼ 12% non-denaturing polyacrylamide gels, silver stained, and photographed.

### Genetic and Deletion Mapping of *Pm21*

Genetic analysis of *Pm21* was performed on an F_2_ population derived from the cross between the resistant line DvRes-1 carrying *Pm21* and the seedling-susceptible line DvSus-1 of *D. villosum*. Chi-squared (χ^2^) test was used to determine the goodness-of-fit of the observed segregation ratio to theoretical Mendelian ratio. Chromosomal deletion analysis was carried out using the susceptible mutant line Y18-S6 obtained from an EMS-induced Yangmai 18 population.

### Comparative Genomics Analysis

The genome sequences of *Brachypodium*, rice and wheat were obtained from the *Brachypodium distachyon* genome assemblies v2.0^1^, the rice genome pseudomolecule release 7^2^, and the IWGSC Sequence Repository^3^, respectively. Genes were predicted using the FGENESH tool^4^ and re-annotated using the BLAST program^5^ and the SMART program^6^.

## Results

### Reactions of *D. villosum* Accessions to *Bgt* Isolate YZ01

**FIGURE 1:**
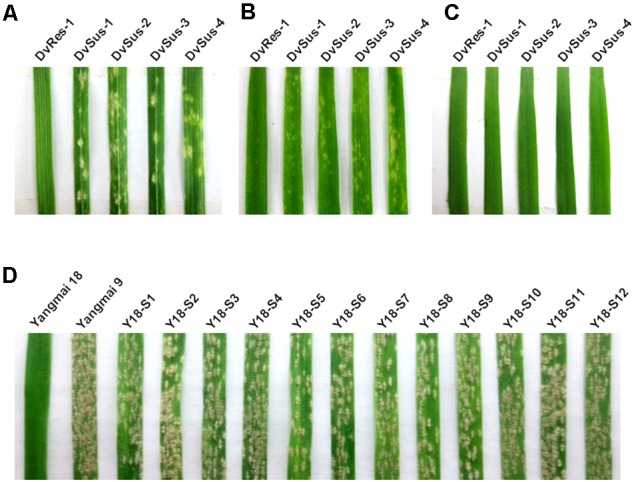
Powdery mildew responses of different *Dasypyrum villosum* lines and Yangmai 18 mutants. **(A–C)** Responses of the resistant *D. villosum* line DvRes-1 and four susceptible lines (DvSus-1 ∼ DvSus-4) to *Bgt* isolate YZ01 at one-leaf stage **(A)**, two-leaf stage **(B)** and three-leaf stage **(C)**, respectively. **(D)** Responses of 12 independent Yangmai 18 mutants (Y18-S1 ∼ Y18-S12) to *Bgt* isolate YZ01 at two-leaf stage, using Yangmai 18 and Yangmai 9 as the resistant and susceptible controls, respectively.

A total of 110 *D. villosum* accessions were collected and inoculated with *Bgt* YZ01 at one-leaf stage. Fortunately, susceptible individuals were identified from four different accessions, and all the other accessions were immune. The susceptible lines obtained were designated as DvSus-1, DvSus-2, DvSus-3, and DvSus-4, respectively (**Table 1**). At different growth stages, powdery mildew responses of the four susceptible *D. villosum* lines were further investigated. All individuals were susceptible at one-leaf stage, but interestingly, an unknown complex resistance gradually increased at two-leaf stage, and fully expressed from three-leaf stage to adult stage (**Figures 1A–C**).

### Development of Polymorphic Markers at Different Ploidy Levels

To develop polymorphic markers between the resistant line DvRes-1 and the susceptible line DvSus-1, a total of 54 6VS-specific markers reported previously (Qi et al., 2010; Chen et al., 2013; He et al., 2016) were tested. Among them, however, only four markers, Xcfe164, 6VS-11, 6VS-25, and 6VS-30, showed polymorphisms between DvRes-1 and DvSus-1. To get more markers for mapping *Pm21*, the CISP-IS strategy based on comparative genomics were applied and 21 polymorphic markers were further developed (Supplementary Table S1), among which, six were single nucleotide polymorphism (SNP) markers. Taken together, a total of 25 markers were recruited for genetic mapping of *Pm21* in diploid *D. villosum*. Using the CISP-IS strategy, another seven 6VS-specific markers were newly developed here and used for physical mapping of *Pm21* in hexaploid wheat (Supplementary Table S2).

### Fine Genetic Mapping of *Pm21*

For genetic mapping of *Pm21* gene, a total of 10,536 F_2_ individuals derived from the cross between the resistant DvRes-1 and the susceptible DvSus-1 were evaluated for their resistance to powdery mildew at one-leaf stage. The result showed that 8,147 were resistant while 2,389 were susceptible, resulting in a resistance-to-susceptible ratio as 3.41:1 that is significantly higher than 3:1, the theoretical Mendelian segregation ratio (χ^2^ = 30.500, *P* < 0.01). Genotyping of F_2_ individuals using the co-segregated marker 6VS-09.4b indicated that the ratio of 2,492 resistant homozygotes, 5,655 heterozygotes, and 2,389 susceptible homozygotes is 1.04:2.37:1, not in accordance with a 1:2:1 ratio (χ^2^ = 59.066, *P <* 0.01).

**FIGURE 2:**
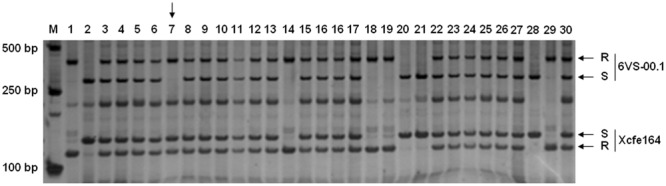
PCR amplification patterns in the F_2_ population using a double-PCR system with the markers 6VS-00.1 and Xcfe164. M: DNA marker DL2000; 1: the resistant parent DvRes-1; 2: the susceptible parent DvSus-1; 3–30: different F_2_ plants. R and S represent the DNA bands that were same to the resistant and susceptible parents, respectively. The vertical *arrow* indicates the recombinant individual.

**FIGURE 3:**
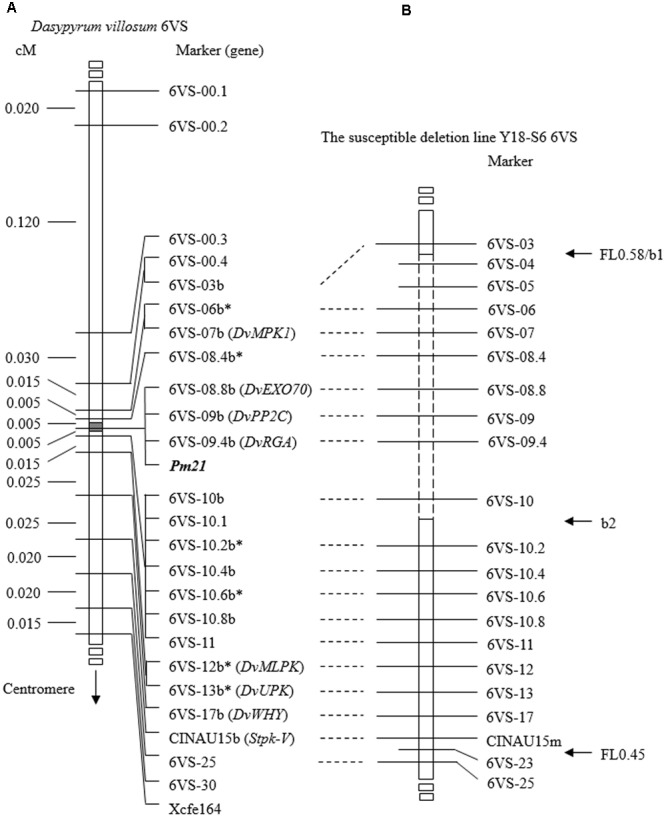
Genetic and physical mapping of *Pm21*. **(A)** Genetic map of *Pm21* and 6VS markers obtained by using the F_2_ population derived from the cross between the resistant *D. villosum* DvRes-1 and the susceptible DvSus-1. The gray block on the chromosome 6VS denotes the genetic interval carrying *Pm21*. All markers used here were derived from genes based on comparative genomics among wheat crops and *Brachypodium* and designated according to the gene order in *Brachypodium*. The DNA markers marked by b and the corresponding ones were developed from the same gene. The asterisks indicate single nucleotide polymorphism (SNP) markers. The genes in brackets were reported previously (Cao et al., 2011; He et al., 2016) or first named in this study. **(B)** Physical map of *Pm21* obtained by using the susceptible deletion line Y18-S6. CINAU15m was a co-dominant marker newly developed from *Stpk-V* gene in this study. The 6VS chromosome breakpoints b1 and b2 in Y18-S6 as well as the bin FL0.45–0.58 are pointed by horizontal *arrows*. The dashed box represents the chromosomal deletion region in Y18-S6. The vertical *arrow* shows the direction of the centromere of 6V.

**FIGURE 4:**
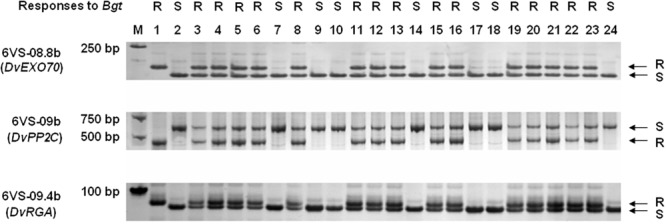
PCR amplification patterns of the co-segregated markers 6VS-08.8b, 6VS-09b, and 6VS-09.4b, corresponding to the genes *DvEXO70, DvPP2C*, and *DvRGA*, respectively. 1: the resistant parent DvRes-1; 2: the susceptible parent DvSus-1; 3–24: different recombinants. Powdery mildew responses were showed above the corresponding parents and recombinants. The *arrows* indicate the DNA bands that were same to the resistant (R) or susceptible (S) parents.

To accelerate screening for recombinants, a double-PCR system was conducted using two markers 6VS-00.1 and Xcfe164, respectively, located on the distal and paracentric regions of 6VS (**Figure 2**). Sixty-four recombinants were identified from the F_2_ population, indicating that the total genetic distance between the two markers was approximately 0.30 cM. Subsequently, a set of 25 markers were used to genotyping these recombinants. In combination with powdery mildew resistance evaluation, *Pm21* was finely mapped into a 0.01-cM interval defined by the markers 6VS-08.4b and 6VS-10b (**Figure 3A**). Furthermore, three genic markers (6VS-08.8b, 6VS-09b and 6VS-09.4b) were found to co-segregate with *Pm21* (**Figure 4**).

### Physical Mapping of *Pm21*

**FIGURE 5:**
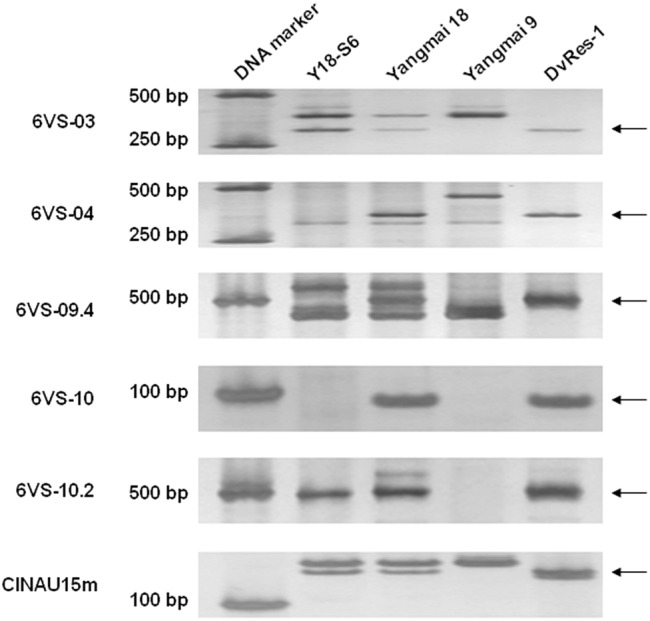
PCR amplification patterns of six representative markers (6VS-03, 6VS-04, 6VS-09.4, 6VS-10, 6VS-10.2, and CINAU15m) used for physical mapping of *Pm21*. The DNA templates used here are successively the susceptible deletion line Y18-S6, Yangmai 18, Yangmai 9, and the resistant *D. villosum* line DvRes-1. The 6VS-specific DNA bands are pointed by *arrows*.

From 1,216 EMS-induced M_2_ families of Yangmai 18 carrying *Pm21*, a total of 12 independent mutant lines highly susceptible to powdery mildew were screened out (Y18-S1 ∼ Y18-S12) (**Figure 1D**). Among them, the susceptible mutant line Y18-S6 was characterized as a chromosomal deletion involving the *Pm21* locus. Molecular analysis revealed the chromosomal breakpoints b1 and b2 in Y18-S6 were closely flanked by the markers 6VS-03 and 6VS-04, 6VS-10 and 6VS-10.2, respectively (**Figures 3B, 5**). Interestingly, the breakpoint b1 in Y18-S6 was close to that in the deletion line del.6VS-1 (FL0.58) reported previously (He et al., 2016). Physical mapping also proved that the deleted chromosomal segment in Y18-S6 spanned the genetic interval carrying *Pm21*.

### Comparative Mapping of *Pm21*

**FIGURE 6:**
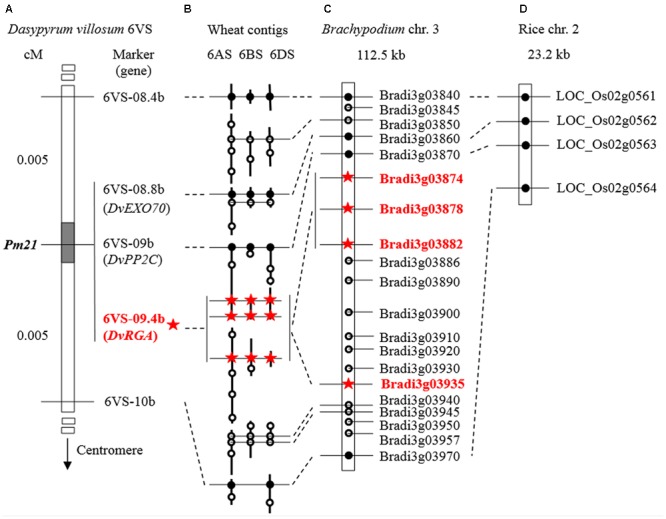
Comparative mapping of *Pm21*. **(A)** Genetic map (partial) using the F_2_ population derived from the cross between the resistant *D. villosum* line DvRes-1 and the susceptible line DvSus-1. The dark region on the chromosome 6VS demonstrates the genetic interval carrying *Pm21*. The vertical *arrow* shows the direction of the centromere of 6V. The complete genetic map can be found in **Figure 3A**. **(B–D)** Comparative maps of the *Pm21* locus among *D. villosum* 6VS, wheat 6AS, 6BS, and 6DS **(B)**, the short arms of *Brachypodium* chromosome 3 **(C)** and rice chromosome 2 **(D)**. The homologous wheat contigs of genes of interest were obtained and annotated by using the BLAST, FGENESH, and SMART programs (**Table 2**). All genes from *Brachypodium* and rice are adopted according to the annotations of the corresponding genomes except the RGA Bradi3g03935 in *Brachypodium* that was re-annotated in this study. The solid circles and hollow circles indicate conserved genes and unconserved genes among different genomes, respectively. The orthologous RGAs among *D. villosum*, wheat and *Brachypodium* are marked by red stars.

The orthologs of the corresponding genes of the flanking markers 6VS-08.4b and 6VS-10b were Bradi3g03840 (2574344–2579921) and Bradi3g03970 (2692381–2693197) in *Brachypodium*, LOC_Os02g05610 (2728848–2734454) and LOC_Os02g05640 (2757693–2758714) in rice, respectively. Hence, the orthologous regions of the *Pm21* locus were narrowed to a 112.5 kb genomic region harboring 18 predicted genes in *Brachypodium*, and a 23.2 kb region harboring two predicted genes in rice. In the orthologous regions, two genes were shared by *Brachypodium* and rice (**Figure 6** and **Table 2**) and used to develop the DNA markers 6VS-08.8b and 6VS-09b. Both of the markers were tested to co-segregate with *Pm21* in the F_2_ population.

**Table 2:** Gene annotation in *Brachypodium*, rice and wheat orthologous regions of the *Pm21* locus.

*Brachypodium*	Rice	Wheat	Gene annotation
Bradi3g03845			Retrotransposable element protein
Bradi3g03850		6AS_contigs_4399884	Eukaryotic translation
		6BS_contigs_2953789	initiation factor
		6DS_contigs_2117578	
Bradi3g03860	LOC_Os02g05620	6AS_contigs_4431592	Exocyst complex subunit
		6BS_contigs_2953283	EXO70-like protein
		6DS_contigs_2092656	
Bradi3g03870	LOC_Os02g05630	6AS_contigs_4363243	Serine/threonine protein
		6BS_contigs_2962596	phosphatase 2C
		6DS_contigs_2093935	
Bradi3g03874^a^		Six contigs^a^	Disease resistance protein
Bradi3g03878^a^		Six contigs^a^	Disease resistance protein
Bradi3g03882^a^		Six contigs^a^	Disease resistance protein
Bradi3g03886			Unknown protein
Bradi3g03890			Unknown protein
Bradi3g03900			Unknown protein
Bradi3g03910			Cytochrome P450
Bradi3g03920			Unknown protein
Bradi3g03930			Poly(A) polymerase
Bradi3g03935^a^		Six contigs^a^	Disease resistance protein
Bradi3g03940		6AS_contigs_4428294	Photosystem II protein J
		6BS_contigs_2926507	
		6DS_contigs_2114667	
Bradi3g03945		6AS_contigs_4428294	Photosystem II
		6BS_contigs_2926507	cytochrome b559 alpha
		6DS_contigs_2114667	subunit
Bradi3g03950			Unknown protein
Bradi3g03957		6AS_contigs_4431958	Polyubiquitin
		6BS_contigs_2953283	
		6DS_contigs_2081863	

*^a^Four RGAs (Bradi3g03874, Bradi3g03878, Bradi3g03882, and Bradi3g03935) in *Brachypodium* are highly homologous with six contigs (6AS_contigs_4363243, 6AS_contigs_4428238, 6BS_contigs_3017519, 6BS_contigs_2958212, 6DS_contigs_2091261, and 6DS_contigs_1402211) on the short arms of wheat homologous group 6.*

Comparative analysis also revealed a conserved resistance gene analog (RGA) locus between *Brachypodium* and wheat orthologous regions (**Figure 6**). In *Brachypodium*, four highly homologous RGAs, Bradi3g03874, Bradi3g03878, Bradi3g03882, and Bradi3g03935, were identified. Among them, Bradi3g03874, Bradi3g03878, and Bradi3g03882 were tandemly aligned as a cluster, and Bradi3g03935 were segregated by other six genes. In wheat, the orthologous RGA loci were also located in the contigs on 6AS, 6BS, and 6DS, and each locus harbored three RGAs at least. According to the conserved locus, the RGA markers 6VS-09.4 and 6VS-09.4b was developed. Physical mapping showed that 6VS-09.4 was lost in the susceptible deletion line Y18-S6, whereas genetic analysis demonstrated that 6VS-09.4b co-segregated with *Pm21* in the F_2_ population.

## Discussion

The powdery mildew resistance gene *Pm21* was previously mapped to the physical bin FL0.45–0.58 of the chromosome 6VS (Cao et al., 2011; Chen et al., 2013). In our previous study, the orthologous regions of the bin FL0.45–0.58 in *Brachypodium* and rice were comparatively mapped (He et al., 2016). In the present study, we carried out physical mapping of *Pm21* by using the susceptible deletion line Y18-S6 obtained from the Yangmai 18 population induced by EMS. We also successfully performed genetic mapping of *Pm21* in diploid *D. villosum*, which was mainly attributed to the finding of *D. villosum* lines seedling-susceptible to wheat powdery mildew. Qi et al. (1998) observed that wheat-*D. villosum* disomic addition line DA6V#1 (Sears, 1953) was susceptible to powdery mildew. However, due to the utilization of colchicine in the incorporation of alien genome by Sears, it is difficult to explain whether the variation came from colchicine treatment or natural variation of the alien parent *D. villosum*. Nevertheless, it remains the possibility to mine susceptible *D. villosum* from natural populations. Thereafter, Qi et al. (1998) screened 46 *D. villosum* accessions but none was susceptible.

We also did not find any *D. villosum* accession susceptible to powdery mildew through the whole growth period. In this study, all the four susceptible *D. villosum* lines displayed complex resistance to wheat powdery mildew, which fully expressed since three-leaf stage. It’s now known to us that powdery mildew resistance of *D. villosum* comes from multi-gene effects, in which, the gene on 6VS carry out an immunologic mechanism different from the others, such as *Pm55*, an adult plant resistance (APR) gene newly found (Zhang et al., 2016). Therefore, to eliminate the background noise, we have to evaluate the *Pm21*-mediated resistance reaction at a very early stage (one-leaf stage). Furthermore, because *Pm21* is immune to all *Bgt* races (Chen et al., 1995), problems probably resulted from race-specific resistance conferred by other genes in background should be avoided in resistance evaluation. Hence, the isolate *Bgt* YZ01 with high virulence was used, to which, only wheat varieties containing *Pm21* or *PmV* (Li et al., 2005; Zeng et al., 2005) were immune. Additionally, *D. villosum* is a cross-pollinated species, which is adverse to forming homozygote of the recessive mutant gene (Hartfield and Glémin, 2014). So, the susceptible homozygotes account for a very small proportion naturally (2 ∼ 5%) as our observation. As a result, susceptible *D. villosum* is difficult to obtain. But fortunately, in this study, we successfully found susceptible individuals from 4 of 110 *D. villosum* accessions.

Based on the discovery of susceptible *D. villosum* resources, genetic mapping of *Pm21* was fulfilled. By double-PCR program, 64 recombinants were identified from 10,536 F_2_ plants, indicating that the total genetic distance of the interval flanked by the markers 6VS-00.1 and Xcfe164 was only about 0.30 cM. It suggested that there exists recombination suppression between two 6VS chromosomes with different origins. The observation given by Qi et al. (1998) also showed that, in wheat background, the two alien 6V chromosomes could not pair normally in pollen mother cells of hybrids between the resistant DA6V#2 (Hyde, 1953) and the susceptible DA6V#1 (Sears, 1953). It is not clear whether recombination suppression observed here is related to low pairing of two different 6V chromosomes in *D. villosum*. In addition, the ratio of resistant and susceptible homozygotes (1.04:1) in the F_2_ population was fit for the ratio 1:1 (χ^2^ = 2.174, *P* > 0.01), suggesting that the male and female gametes have no obvious difference in viability and transmission. However, unexpectedly, the ratio of heterozygotes (53.6%) was higher than the theoretic ratio (50%), indicating that heterozygotes could survive more easily, which might be due to the heterosis in cross-pollinated *D. villosum*.

In the previous studies, eight genes, including *Stpk-V, DvMPK1, DvMLPK, DvUPK, DvPSYR1, DvPP2C, DvGATA*, and *DvWHY*, have been mapped to the bin FL0.45–0.58 carrying *Pm21* and confirmed to be required by the *Pm21* resistance. However, silencing of these genes mediated by barley stripe mosaic virus (BSMV) could not lead to macroscopic symptom of powdery mildew (Cao et al., 2011; He et al., 2016). It is suggested that these genes play roles in complex *Pm21* resistance pathways but none of them is the *Pm21* gene itself. Interestingly, overexpression of *Stpk-V* can confer high resistance to powdery mildew in transgenic wheat (Cao et al., 2011). Hence, these genes might have potential to be used to transgenic breeding for control of wheat powdery mildew. Furthermore, revealing their functions and mechanisms could contribute to understanding disease resistance pathways mediated by *Pm21*. Among the above genes, only *DvPP2C* (6VS-09b), encoding a serine/threonine protein phosphatase 2C, was confirmed to be located in the genetic interval carrying *Pm21* by physical, genetic and comparative analyses in this study. In addition, another gene *DvEXO70* (the corresponding markers 6VS-08.8 and 6VS-08.8b), encoding an exocyst complex subunit EXO70-like protein, appears in the *Pm21* involved interval; however, whether it plays a role in the *Pm21* resistance remains unclear.

Although disease resistance genes evolve rapidly and have limited collinearity in cereals (Keller et al., 2005), a conserved RGA locus was observed in *Brachypodium* and wheat orthologous regions of the genetic interval of *Pm21*. The members in this RGA locus encode typical coiled-coil, nucleotide-binding site, leucine-rich repeat (CC-NBS-LRR) proteins that are the major class of plant disease resistance proteins (Gururani et al., 2012). Both physical mapping and genetic mapping demonstrated that the corresponding RGA markers 6VS-09.4 and 6VS-09.4b were located in the *Pm21* locus. It suggested that this co-segregated RGA locus could be considered as an important candidate of the *Pm21* locus. However, due to large genome size, it would be still a great challenge to directly perform map-based cloning of *Pm21* in *D. villosum*. Recently, a fast cloning method using mutagenesis and next-generation sequencing (NGS) has been successfully utilized to identify the stem rust resistance genes *Sr22* and *Sr45* (Steuernagel et al., 2016) and the powdery mildew resistance gene *Pm2* (Sánchez-Martín et al., 2016) in wheat. In this study, 11 independent susceptible wheat mutants, not involving in chromosomal deletion, could be used to find the exact sequence of *Pm21*, and further researches are in progress.

## Author Contributions

HH and TB conceived and designed the experiments. HH screened the resource of *D. villosum*. YJ and SZ developed DNA markers. YJ, BL, RZ, and ZJ performed genetic mapping. HH and TB performed physical mapping. HH and SZ analyzed the data and wrote the paper. HH and TB revised the paper.

## Conflict of Interest Statement

The authors declare that the research was conducted in the absence of any commercial or financial relationships that could be construed as a potential conflict of interest.

## Footnotes

1 http://www.brachypodium.org

2 http://rice.plantbiology.msu.edu

3 http://wheat-urgi.versailles.inra.fr

4 http://linux1.softberry.com

5 http://blast.ncbi.nlm.nih.gov/Blast.cgi

6 http://smart.embl-heidelberg.de
