# Supplementary material for: Genetic, Physical and Comparative Mapping of the Powdery Mildew Resistance Gene Pm21 Originating from Dasypyrum villosum
Source: Front Plant Sci. 2017 Nov 7;8:1914. doi: 10.3389/fpls.2017.01914 (PMC5681962; doi:10.3389/fpls.2017.01914)
Supplement: Supplementary file 1 [file Table_1.DOC]

**Table S1 | DNA markers used for genetic mapping of *Pm21*.** F: forward primer; R: reverse primer. The DNA markers marked with b were the modified versions of the corresponding ones are suitable for genetic mapping in diploid *D. villosom*. The markers with asterisks are single nucleotide polymorphisms (SNPs). The EST-SSR marker Xcfe164 was reported by Qi et al (2010).

| DNA marker | Primer sequence (5’→3’) |
| --- | --- |
| 6VS-00.1 | F: GGACATGACCCATCTGTTTGTG |
|  | R: GCAATGGTCTGTCCCCTGACA |
| 6VS-00.2 | F: CGGGAGAGGGCTTCCATTCA |
|  | R: GCTTGCCCAATTACCTCGTCT |
| 6VS-00.3 | F: GATGCCCTGGAACTGTTGTATCAG |
|  | R: GTGAATGAAGTCCAAACCTTTGCA |
| 6VS-00.4 | F: TGGTGGTTCACTGCTTCTGGTAC |
|  | R: CTTGGTGCTGTTCTGATCTCTATGTC |
| 6VS-03b | F: GCTGCTTCTGTACTCTTTGACTTGGA |
|  | R: CCAATCGTCCAGGTGCTCTTGCA |
| 6VS-06b* | F: CCTAACACCCTTGGAAGTTGGA |
|  | R: CAGTCTTTAGCATGTGCTTGCAT |
| 6VS-07b | F: CGAGCAGGCACAACCTTCAAG |
|  | R: GGTCGCCGCAATACACACTGT |
| 6VS-08.4b* | F: CCCTCCAACACTACTAGTCCCTGT |
|  | R: CAGTGACTTCTTCCTCAGGAGCATC |
| 6VS-08.8b | F: TGCCTGAGTGCAAGGACTGTC |
|  | R: CACCTCCAACACAAGTGCTATAC |
| 6VS-09b | F: GCAGGATAGAAATCCATGGCAT |
|  | R: CAACATCATCCACTCTTGTCTCGTAG |
| 6VS-09.4b | F: GCTTGTCGGAACCGAAGATG |
|  | R: CTGACTTCTAATTCCACATCAATCA |
| 6VS-10b | F: CGTCCTCTCTCACGTACGTCCA |
|  | R: CAGACTCTTCTGACCCGTGCA |
| 6VS-10.1 | F: GGGATATGAAACTTTGTATGTGCCT |
|  | R: GACATGTGGAGTTGCCAGTCGT |
| 6VS-10.2b* | F: GACGTGGCGGTGTTAGGTCA |
|  | R: GCACGCTCCTCCGTTGTCA |
| 6VS-10.4b | F: CTGAACTGTGAACCTACTCTAACGCT |
|  | R: AAGGATCGCAGCATACACACTG |
| 6VS-10.6b* | F: CGTCTCCATCCCACTATCCTAGTAC |
|  | R: CAACCTCTTCGCCCTTCACGA |
| 6VS-10.8b | F: CTGATTTAGTGGAACATAGAGCAGG |
|  | R: CCCTGATTTGACACAGATGGTGA |
| 6VS-11 | F: TCCAGGAGAATGGCCAGTCA |
|  | R: GGCGGACATACCTGGGATC |
| 6VS-12b* | F: GGATTTGTCCAGCAATGATCTGAG |
|  | R: CTGATCCATCTTCAGGACCAGTAG |
| 6VS-13b* | F: GCACTGAAATGCTGCGAGCTG |
|  | R: GAAAATGATCCTGCCTTCGCACT |
| 6VS-17b | F: CCTGTAACAAAAGCTGAATTTGCAGT |
|  | R: GCTCGAACCTAGGCTTGCTAGCT |
| CINAU15b | F: CACACGGGTTGCAGGAACATTG |
|  | R: ACTCAGGAGCCAAGTAACCTCTGTGA |
| 6VS-25 | F: TAAGCTTCTAGATAGGTTGAGCTATGACA |
|  | R: TGCCAGGTCACGCAACGAC |
| 6VS-30 | F: GAACCTACCGCTGGATGGCA |
|  | R: CAGCAGAAGGTTGTTAATCAGATGCA |
| Xcfe164 | F: GGAACCCAGGCGAGGTAG |
|  | R: AGCCTAGCAGCATAGCATCC |
