# Supplementary material for: Genetic, Physical and Comparative Mapping of the Powdery Mildew Resistance Gene Pm21 Originating from Dasypyrum villosum
Source: Front Plant Sci. 2017 Nov 7;8:1914. doi: 10.3389/fpls.2017.01914 (PMC5681962; doi:10.3389/fpls.2017.01914)
Supplement: Supplementary file 2 [file Table_2.DOC]

**Table S2 | DNA markers used for physical mapping of *Pm21*.** F: forward primer; R: reverse primer. The DNA markers are 6VS-specific and suitable for physical mapping in hexaploid wheat. The marker CINAU15m was newly developed from the *Stpk-V* gene in this study. The others unlisted were reported in our previous work (He et al. 2016).

| DNA marker | Primer sequence (5’→3’) |
| --- | --- |
| 6VS-08.4 | F: AGGCGAAAATGGCAATGCA |
|  | R: AGTGATCCAAATGCCCCAGGT |
| 6VS-08.8 | F: GTGTTAAGTCTACTGTATCTGGTTTGA |
|  | R: CAAGCATATTGGTTCAAATTCAC |
| 6VS-09.4 | F: AATTGCATTGATGACTTCATGCGAG |
|  | R: CTCCAAATCCAACAATAGCCACCA |
| 6VS-10.2 | F: CGTCAAATAAGAACATCCAACGTG |
|  | R: TATGGGGCCCTTCGCCTT |
| 6VS-10.4 | F: CATAATTTATTCATCATCGCCAACT |
|  | R: GCGTGCTTCTGGGCGAGGTA |
| 6VS-10.6 | F: CGTCTCCATCCCACTATCCTAGTAC |
|  | R: CAACCTCTTCGCCCTTCACGA |
| 6VS-10.8 | F: TCGATTGGAACTGATTTAGTGGAAC |
|  | R: ACCCTGAGCTCGTCTCCCTCTA |
| CINAU15m | F: TGTTTCGTAGGAGAAGTTAGAGGCA |
|  | R: ACTCAGGAGCCAAGTAACCTCTGTGA |
